# The Effectiveness of Orofacial Pain Therapy in Indonesia: A Cross-Sectional Study

**DOI:** 10.1155/2018/6078457

**Published:** 2018-07-18

**Authors:** T. Maulina, G. Yubiliana, R. Rikmasari

**Affiliations:** ^1^Oral Surgery Department, Faculty of Dentistry, Universitas Padjadjaran, Bandung, Indonesia; ^2^Community Dental Health Department, Faculty of Dentistry, Universitas Padjadjaran, Bandung, Indonesia; ^3^Prosthodontic Department, Faculty of Dentistry, Universitas Padjadjaran, Bandung, Indonesia

## Abstract

**Objective(s):**

As the most complained oral problems in Indonesia, the therapy of orofacial pain has to be constantly evaluated. The objective of the current study was to evaluate the effectiveness of orofacial pain therapy in Indonesia.

**Method(s):**

This study recruited 5412 (3816 female; 1596 male) participants from 27 districts in West Java province. Half of the participants (2714) were recruited from those who were treated at community health centers whilst the rest were those who were treated at private dental clinics. A Likert-scale questionnaire that consists of nine questions that were divided to three subsections was used. The first subsection of the questionnaire evaluated the participants' post-therapy basic oral functions (three questions), and the second part evaluated the participants' post-therapy pain intensity and frequency (three questions), whilst the last part evaluated the participants' post-therapy activities (three questions). All data were then cross-tabulated and correlated by using Spearman correlation.

**Result:**

The current study revealed that out of 5412 participants, 4023 (74.33%) participants claimed that the therapy has enabled them to perform their work activity as usual, whilst 2576 (59.2%) claimed that the therapy has decreased the intensity of the pain moderately. A significant (p < 0.01) correlation (r = 0.1) between the type of dental facility visited and the total score of the therapy effectiveness was revealed.

**Conclusion:**

The therapy of orofacial pain in Indonesian sample was proven to be effective. Further study evaluating the reasons underlying the current results is of importance.

## 1. Introduction

As one of the most complained dental problems, orofacial pain has affected its sufferer at so many levels. Not to mention that almost every epidemiological study about the impact of orofacial pain revealed the detrimental effect of orofacial pain in daily activities, work activities, and social activities [1–3]. Aside from the impact on life activities, orofacial pain was also reported for its impact on oral health-related quality of life [4, 5] due to pain and impaired oral function activities, namely, mastication, mouth opening, and speaking.

In a study conducted by Shueb et al. (2015) about the impact of chronic and acute orofacial pain on 146 patients that suffered from one of the following: temporomandibular disorders (n=41), acute dental pain (n=41), trigeminal neuralgia (n=21), and persistent dentoalveolar pain (n=22) by using the Oral Health Impact Profile (OHIP)-49, it was revealed that all pain disorders showed a significant (p<0.001) impairment compared to the control group (pain-free group, n=21) [6]. Whilst another research conducted by Tjakkes et al. (2010) about the impact of orofacial pain on patients' health-related quality of life (n-95) showed that patients that have been having TMD pain for the period of two to three years showed a significantly worse social and physical functioning compared to those of the general population [7].

Considering the detrimental impact of orofacial pain on the patient's quality of life, assuring that orofacial pain patients are receiving effective therapy is considered to be of importance, as pain elimination will improve patients' oral function, which in return will also improve patient's quality of life. A literature study by Katz (2002) revealed how patients' quality of life is impaired when pain is not effectively treated [8]. Another literature review about the importance of effective pain management by Glowacki (2015) revealed that adequate pain management enhances earlier mobility as well earlier overall recovery, improved quality of life, increased productivity, and decreased cost for patients and the health care system [9].

In regard to the health care system, in Indonesia, there are two types of dental facilities, those that are government facilities and those that are private facilities provided by private parties. In relation to this, a study performed by Al-Hussyeen (2010) about the factors affecting the utilization of dental health services revealed that patients are more likely to use private dental practice services due to its high quality of dental care, and those who utilized the service of dental public clinics found postoperative complications to be the discouraging factor for its future utilization [10]. It is important for every medical/dental to provide an excellent service quality as the service quality of medical/dental facilities will improve the service quality of the whole health care system [11].

Unfortunately, regardless of the impact of orofacial pain in patient's quality of life and the importance of effective therapy for the patients as well as the health care system, our literature study showed no record of previous study(ies) that evaluated the effectiveness of orofacial pain therapy in public and private dental facilities. Therefore, the aim of this study was to evaluate the effectiveness of orofacial pain therapy in Indonesian sample and to compare the effectiveness of the therapy given in a private dental clinic and public dental facility.

## 2. Material and Methods

The current study was conducted at West Java province, Indonesia, and recruited 5412 (3816 female: 1596 male) participants (Table 1) that were gathered from all 27 districts and cities located in the province. From every city or district, every private practice and community health centers were listed and were picked randomly by using an envelope that contained the name of the dental facility. All field researchers then visited the dental facilities and recruited 200 participants, of which (at least) 100 participants were those who were treated at the community health centers, and (at least) 100 participants were those who were treated at a private dental practice or clinic. A sample size calculation has been adequately performed and was performed by an experienced statistician.

All recruited participants should fulfill the following inclusion criteria: age between 18 and 45 years old, currently having an occupation, receiving the (pharmacological and nonpharmacological) therapy of orofacial pain (surgery excluded) they had prior to the survey (within the period of six months), and having been living in the province for at least two years. Ten calibrated interviewers interviewed the participants by using a validated questionnaire. In order to avoid any potential fatigue that might affect the results of the interview, one interviewer can only interview ten participants per day.

Prior to the start of the study all participants signed an informed consent regarding their participation in the study. An ethical clearance was obtained from the Profession and Research Ethics Committee of Medical Committee Faculty of Dentistry Dental Hospital Padjadjaran University. To confirm, every procedure and ethical aspect of the current research have been conducted in full accordance with the World Medical Association Declaration of Helsinki. All participants gave their written consent for their participation in the current study as well as the usage of any of their photograph(s) that was taken during the study that was related to the study procedure, in all possible publications related to the study.

A validated questionnaire (Table 2) with a total of ten questions that consists of one question about the type of orofacial pain experienced and nine questions that evaluated the effectiveness of the therapy and divided into three main sections was used in the current study (Cronbach's Alpha: 0.824; r value(s) > 0.312; p value(s) <0.05). The types of orofacial pain experienced were toothache, pain in the jaw joint/s, pain in area just in front of the ear/s, pain in or around the eyes, pain when opening the mouth wide, shooting pains in the jaw joint when chewing food, pain and in around the temples, tenderness of muscles at the side of the face, and a prolonged burning sensation in the tongue or other parts of the mouth.

The first section of the questionnaire comprised three questions that were composed for the purpose of oral function evaluation whilst the questions in the second section were composed to evaluate pain intensity as well as pain occurrences. For the pain occurrences question (during the interview) the interviewer gave the participants a standardized range for each answer (for example,** not at all**: if the participants think that they are still having the pain just as often as they used to prior to the therapy;** a little bit**: if the pain occurs about 3/4 of the rate that they used to;** moderately**: if the pain occurs about 1/2 of the rate that they used to; and** a lot**: if the pain occurs about 1/4 of the rate that they used to). The last part of the questionnaire evaluated the patients' ability in performing their work activity, social activity, and daily activity. Every question was provided with four Likert-scale option type of answer, which were “not at all”, “a little bit”, “moderately”, and “a lot”. After the questionnaire was completed, all recorded answers were scored.

The scoring system was as follows: “not at all” was given a score of “0”, “a little bit” was scored as “1”, “moderately” was scored as “2”, and “a lot” was scored as “3”. Based on the scoring system, a total score that ranged between 0 and 9 was considered as a reflection of poor effectiveness of the therapy, and 10 to 18 reflected moderate effectiveness of the therapy, whilst a score equal to or more than 19 reflected a high level of effectiveness. All gathered data were analyzed by using cross-tabulation analysis and A Chi-square test to evaluate any significant difference. A correlation between variables was conducted by using the Spearman Rank correlation test.

## 3. Results

The first data evaluation of the current study revealed the distribution of participants for each of orofacial pain being investigated in the current study (Table 1). Consistent with our previous research, the most experienced orofacial pain was odontogenic origin (toothache, 53.43%), whilst the least experienced was pain around the eyes with only 62 participants (1.15%) experiencing this particular type of orofacial pain. Based on the analysis about the orofacial pain therapy effectiveness generated in the current study, it was revealed that the average score of the therapy effectiveness was 22.45, with 27 being the highest score possible.

**Figure 1:**
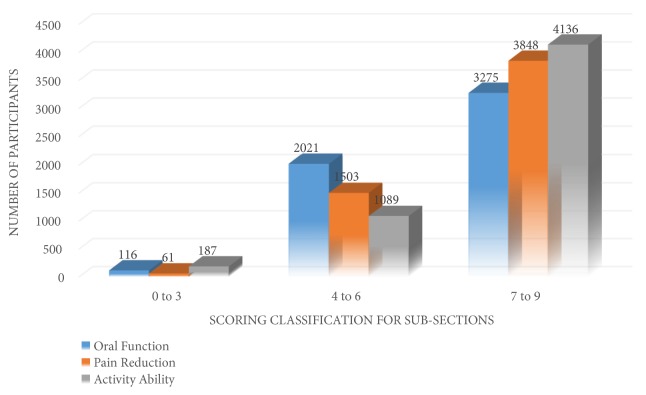
Participants distribution based on the subsection score of the questionnaire.

Aside from the total score of the questionnaire, a scoring evaluation for each subsection of the questionnaire was also conducted. The result of the analysis showed that the mean score for the first subsection (oral function) was 7.1, the mean score for the second subsection (pain reduction) was 7.3, and the mean score for subsection 3 (activity performance) was 8.1. When the participants were asked about their ability in performing their usual work, social, and daily activity after the therapy was given it was revealed that most of the participants were in the “7 to 9” level of scoring (Figure 1).

**Figure 2:**
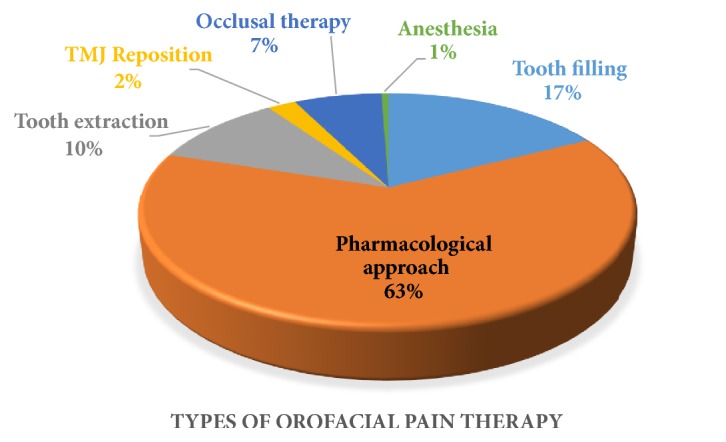
Participants distribution based on the type of orofacial pain therapy.

Aside from the scoring of the questionnaire, the types of the therapy provided by both dental facilities were also recorded (Figure 2). An analysis about the level of improvement experienced by the participants revealed that the participants experienced the most moderate level of improvement when it comes to chewing improvement (2859; 52.8%) as well as pain occurrences reduction (2576; 47.6%) (Table 3). As for high level of improvement, participants claimed that the highest level of improvement was experienced when it comes to their ability in performing their work activity (4023; 74.3%) and daily activity (4081; 75.4%) as per usual.

Another analysis performed in the current study was the correlation between the sex of the participants and the total score of the questionnaire. There was a significant (p<0.01) positive correlation (r=0.1) between the sex of the participants and the total score. It was revealed that more female (3252; 85.2%) participants claimed that the therapy has improved their oral function better, reduced pain intensity and occurrences more effectively, and enabled them to perform their activities better compared to the male (1305; 82.17%) participants (Table 4). As for age, there was a significant (p<0.01) positive correlation (r=0.1) found between age and the level of therapy effectiveness. It was revealed that more participants (2903, 85.8%) who aged between 31 and 45 years had higher therapy effectiveness score compared to those who are aged 18 to 30 years (1654; 81.5%).

A significant (p<0.01) positive correlation (r=0.1) was also found between the type of dental facility (community health centers or private dental clinics) and the total score of the questionnaire. The therapy being given in the private dental clinics seemed to have more participants (2326; 86.2%) with the total score of ≥19 compared to those who visited the community health centers (2231; 82.2%). There was no significant difference found between age and the type of dental facility that the participants have visited when it comes to preferred dental facility type.

## 4. Discussion

On the survey about the prevalence of orofacial pain in West Java province, Indonesia, in 2016, it was revealed that most participants who experienced orofacial pain of any type complaint about the interference they experienced on three basic oral functions: chewing, speaking, and jaw movement (opening and closing). (1) This particular research result was the main consideration for choosing these three oral functions to be evaluated in the current study. As for the evaluation of activities, previous studies have emphasized about the interference of orofacial pain on these activities [12, 13].

The first analysis of the study revealed the mean total score of the questionnaire. The facts that the total mean score was 22.45 and the subsection analysis showed a score as high as 8.1 for the ability to perform activities as per usual were solid indicators of the fact that the orofacial pain therapy given was considered to be highly effective. Considering the high prevalence of orofacial pain in Indonesian sample obtained from previous survey, (2) this particular result is expected to serve as an indicator that the West Java government has used the epidemiological information about the high prevalence of orofacial pain as a scientific background in the planning stage of orofacial pain management and implemented effective measures in reducing the high prevalence. It is important to note how the therapy has highly affected the participant's ability to perform their work activity as usual, especially as we were investigating a productive age group and that interfered work ability and lost productivity have been one of the highlights when it comes to pain impact assessment [14–16] in this particular field and age group.

In the current study, we evaluated the type of orofacial pain therapy being conducted in both types of dental facilities (Figure 2). And like any other type of pain in the human body, there are two types of approach for the treatment of orofacial pain, pharmacological approach and nonpharmacological approach. The pharmacological approach of orofacial pain usually involves analgesic, anti-inflammation nonsteroids, corticosteroids, benzodiapine, muscle relaxants, anticonvulsants, and antidepressants [17–20]. In this study, it was revealed that pharmacological approach was the most common therapy being given to orofacial pain patients, which might be due to the fact that toothache or dental pain was the most common type of orofacial pain experienced by the participant, and therefore, the first measure taken to manage the pain was by analgesic consumption. As for the nonpharmacological approach, it generally consists of patient education, biobehavioral therapy, posture training, mobilization, electrotherapy, ultrasound, iontophoresis, anesthesia, acupuncture, laser therapy, occlusal appliance therapy, and surgery [18, 20] which was in line with the findings of our study (Figure 2). The variability of the course of therapy is, of course, expected to increase the success rate of the management of orofacial pain.

Another result of the current study was the improved oral functions after the therapy, one of which was the chewing function. In a study by Choi et al. (2009) about pain disability on orofacial pain patients, chewing function was found to be one of the most interfered oral functions in orofacial pain patients [21] amongst other interfered oral functions. Other previous studies [22, 23] also reported interfered physical oral functions including chewing function due to orofacial pain or temporomandibular disorders that was accompanied by pain, which in turn has decreased the patients' quality of life. In a study conducted by De Laat et al. (2003) about the impact of different course of therapy on myofascial pain of the masticatory system patients, a decreased pain score after four to six weeks of therapy was revealed, accompanied by a decreased Mandibular Function Impairment Questionnaire (MFIQ) score, indicating that the pain reduction has improved patients' oral function [24].

In another study conducted by Bennet et al. (2005) about the impact of pharmacological approach on patients with fibromyalgia pain, it was revealed that the pain decreased has also impacted patients' ability to work and to function socially [25]. These previous findings are, therefore, in line with the findings of the current study, whereas reduced pain intensity and improved oral functions (chewing, speaking, as well as mouth opening and closing) were accompanied by improved daily or activities performance.

There are several significant correlations found in the current study, one of which was the significant correlation between the type of dental facilities visited and the overall improved functions being evaluated in the study. It was revealed that more patients who visited private dental facilities and being treated in this particular type of dental facilities claimed that the therapy allowed them to perform their work, daily, and social activities as per usual in the “a lot” category, compared to those who visited community health center. This particular result might indicate the high effectiveness of the therapy as well as the participants' high level of perceived satisfaction when it comes to the quality of the therapy in private dental facilities.

In a study conducted by Hancock et al. (1999) about the perception and experiences of patients who went to private dental facilities compared to those who went to public dental facilities, it was revealed that those who went to private dental facilities claimed that they are more satisfied with the treatment being given, compared to those who went to public dental facilities [26], which is in line with the current results. Despite the difference in the number of participants, there was no significant difference found. This particular result of the current study is in line with previous studies concerning the service being given by private and public dental facilities [27, 28].

The variability of results elaborated above indicates the need of further studies that thoroughly investigates the correlation between different types of dental facility and the effectiveness of the orofacial therapy. Yet, in regard to therapy efficacy, our study has provided scientific information on how orofacial pain patients who visited private dental practice were more satisfied with the therapy compared to those who visited the community health centers. Lastly, in relation to the results of the current study, it is concluded that therapy being given to orofacial patients in West Java province is considered to be highly effective in eliminating orofacial pain, restoring oral function, and improving participants' daily, work, and social activities. It is hoped that the result of the current study can be used by the government of the West Java Province as one of the scientific backgrounds in preparing better health quality service.

## Figures and Tables

**Table 1:** Demographical and clinical characteristics of participants.

**Variables**	**Categories (Number of participants)**								
Sex	Male					Female			
	**1596**					**3816**			

Age	18 – 30 years old					31 – 45 years old			
	**2029**					**3383**			

Type of dental facility	Community Health Center (Puskesmas)					Private Dental Practice / Private Dental Clinic			
	**2714**					**2698**			

Types of orofacial pain^*∗*^	**1**	**2**	**3**	**4**	**5**	**6**	**7**	**8**	**9**
	**2892**	**987**	**88**	**62**	**376**	**688**	**67**	**120**	**132**

^*∗*^Types of orofacial pain: (1) toothache; (2) pain in the jaw joint/s; (3) pain in area just in front of the ear/s; (4) pain in or around the eyes; (5) pain when opening the mouth wide; (6) shooting pains in the jaw joint when chewing food; (7) pain and in around the temples; (8) tenderness of muscles at the side of the face; and (9) a prolonged burning sensation in the tongue or other parts of the mouth.

**Table 2:** Orofacial pain therapy effectiveness questionnaire.

Name:					
Age:					
Sex:					

No	Questions	Level			
		Not at all	A little bit	Moderately	A lot

1	After the therapy, did you feel any improvement on the following functions?				
	(a) Chewing				
	(b) Speaking				
	(c) Closing and Opening the mouth				

2	After the therapy, did you feel that:				
	(a) the intensity of your pain decrease?				
	(b) the frequency of the occurrence of the pain decrease?				
	(c) the pain decrease when you perform certain jaw movement?				

3	After the therapy, were you able to perform the following activities as per usual?				
	(a) Work activity				
	(b) Social activity				
	(c) Daily activity				

**Table 3:** Distribution of participants based on level of improvement.

**No**	**Question type**	**Level of improvement**				**Total**
		Not at all	A little bit	Moderately	A lot	
**1**	Chewing improvement	33	887	**2859**	1633	5412
		(0.6%)	(16.4%)	**(52.8**%**)**	(30.2%)	(100%)

**2**	Speaking improvement	14	251	2194	2953	5412
		(0.3%)	(4.6%)	(40.5%)	(54.6)	(100%)

**3**	Jaw movement improvement	14	379	2162	2857	5412
		(0.3%)	(7.0%)	(39.9%)	(52.8%)	(100%)

**4**	Pain intensity reduction	15	613	**2576**	2208	5412
		(0.3%)	(11.3%)	**(47.6**%**)**	(40.8)	(100%)

**5**	Pain occurrences reduction	9	486	1827	3090	5412
		(0.2%)	(9.0%)	(33.8%)	(57.1%)	(100%)

**6**	Pain on jaw movement reduction	10	320	1766	3316	5412
		(0.2%)	(5.9%)	(32.6%)	(61.3)	(100%)

**7**	Work activity improvement	2	285	1102	**4023**	5412
		(0.0%)	(5.3%)	(20.4%)	**(74.3**%**)**	(100%)

**8**	Social activity improvement	3	328	1183	3898	5412
		(0.1%)	(6.1%)	(21.9)	(72.0%)	(100%)

**9**	Daily activity improvement	3	229	1099	**4081**	5412
		(0.1%)	(4.2%)	(20.3%)	**(75.4**%**)**	(100%)

**Table 4:** Participant distribution based on therapy effectiveness score.

**No**	**Variables**		**Effectiveness Score**		
			Low	Moderate	High
			(0-9)	(10-18)	(**≥**19)
1	**Sex**	Male	3	288	1305
		Female	11	553	3252

2	**Age**	18-30 years old	3	372	1654
		31-45 years old	11	469	2903

3	**Type of dental facilities**	Community	9	474	2231
		Private	5	367	2326

## Data Availability

All data of the current study are available at the database of the researcher. Anyone who is at interest of the data may contact the corresponding author through email correspondence and discuss the possibility of data viewing or further usage.
